# Fuzzy Fault Detection and Band-Conditioned Data Quality Assessment for Sensor Streams: A Continuous Glucose Monitoring Case Study

**DOI:** 10.3390/s26206442

**Published:** 2026-10-12

**Authors:** Juan F. Gaitán-Guerrero, Carmen Martínez-Cruz, Macarena Espinilla, José L. López

**Affiliations:** 1 Department of Computer Science, University of Jaén, Campus Las Lagunillas, 23071 Jaén, Spain; mestevez@ujaen.es (M.E.); llopez@ujaen.es (J.L.L.); 2 Department of Languages and Computer Systems, University of Granada, 18071 Granada, Spain; cmcruz@ugr.es

**Keywords:** data reliability, sensor monitoring, fuzzy logic, linguistic protoforms, uncertainty-aware aggregation, continuous glucose monitoring

## Abstract

Sensor faults can propagate beyond raw measurements and distort the aggregates used to interpret a monitored phenomenon. We propose a domain-agnostic, five-layer fuzzy pipeline that characterizes the reliability of a sensor stream jointly over time and value bands and carries it through to every derived aggregate: (L1) fault tagging of each sample against five fuzzy failure primitives; (L2) fuzzy windowing; (L3) band reliability; (L4) routed repair with propagated reconstruction uncertainty; and (L5) veracity aggregation with linguistic protoforms. An end-to-end acquisition architecture is also specified. The pipeline was instantiated for continuous glucose monitoring (CGM) with clinical knowledge of glucose dynamics and applied to a 14-month recording of an adult with type 1 diabetes (FreeStyle Libre 3, 5-min cadence), of which 294 days contained at least 120 readings. Clean reference days remained essentially unchanged (mean sample quality 0.98–0.99). On the most corrupted day, the estimated time below range fell from 16% to 3%, with a veracity of only 0.14. Over the recording, 38% of the days were too unreliable to phenotype and 5% changed phenotype. Joint time- and band-conditioned reliability assessment thus prevents corrupted sensor data from producing falsely precise clinical conclusions.

## 1. Introduction

Diabetes management increasingly relies on continuous glucose monitoring (CGM). Its minute-by-minute readings are condensed into a small set of aggregates that comprise time in range (TIR), time below and above range (TBR, TAR), mean glucose and the glucose management indicator, which now constitute standard clinical targets and drive therapeutic decisions [1]. Yet, CGM sensors are imperfect: they leave gaps during warm-up, disconnection or compression; they saturate against the lower bound of the assay; they stick at a constant value; and they emit physiologically impossible excursions [2]. These faults are rarely confined to the raw signal. Once undetected, they flow through cleaning, aggregation and reporting and silently distort the very metrics on which treatment rests: a few spurious low readings inflate the TBR and can fabricate an episode of hypoglycaemia, while an unnoticed gap leaves the TIR simply unknowable for part of the day; the metrics themselves, moreover, are known to vary with the sensor model and to carry a systematic bias [3,4].

A central observation motivates this work: the reliability that results from these faults is not a single number attached to a series or a day, but a field defined over two axes: time and glucose range. The same day of data can be perfectly trustworthy for detecting hyperglycemia while being unusable for hypoglycaemia, simply because the corrupting fault falls in the low range during a particular interval. Current practice ignores this structure: the international consensus on CGM interpretation gates analysis on a single crisp criterion—at least 70% of sensor-active time over 14 days [1]—which neither localizes unreliability in time nor conditions it on the glucose range it affects.

Neither these failure modes nor this blind spot, however, are specific to glucose: missing values, anomalous readings, and the silent way they contaminate downstream aggregates recur across environmental sensing [5,6], structural health monitoring [7], wearable physiology and the industrial internet of things (IoT), where sensor faults degrade the service quality of deployed networks [8] and dropouts corrupt the monitoring of offshore installations [9]. Each of these fields has confronted a piece of the same problem under its own name and in isolation from the others.

In biomedical signal processing, the dominant response has been to clean the stream itself: a smart sensor cascade of denoising, enhancement and prediction placed after a commercial CGM device [2,10], driven by a Kalman filter that adapts to a signal-to-noise ratio varying across sensors and individuals [11], with later variants tackling coloured, non-stationary noise or reconstructing corrupted stretches outright [12,13]. A parallel effort detects faults rather than removing them, modeling transient disconnections and compression artifacts [14] and flagging sensor failure through statistical or clustering criteria [15,16,17]; in wearable monitoring, the same idea takes the form of signal-quality indices that grade each segment and route the processing that follows [18,19]. Such methods work at the sample level and serve real-time use, i.e., alarms, dosing, and control, but they do not carry a judgment of reliability forward to the aggregates that summarize a day, nor put it in a form a clinician can read.

A largely separate tradition formalizes data quality in the abstract, as a set of dimensions such as completeness, accuracy, consistency, timeliness and, more recently, as a context-dependent notion of “fitness for use” [20], with consolidated metric sets and frameworks that add reliability flags and interpolation over the raw stream across the IoT, wearables, environmental sensing and manufacturing [21,22,23,24,25]. This offers a useful vocabulary, yet stops short of a characterization that is resolved in time, conditioned on the value range, and tied to the specific quantity being computed. Where data are missing or noisy, the classical remedies such as interpolation, Kalman or Gaussian-process smoothing, multiple imputation, and their fuzzy-rough variants [26] remain in active use across monitoring domains, from multiple interpolation of long environmental records [27] to generative reconstruction of multivariate sensor streams [28], and have lately been refined to return a variance alongside each reconstructed value, making explicit how much was inferred [29,30,31]. Closest to our own concern, Hosseini and Moeini [32] derive a reliability index and use it to complete gaps in a sensor record; the index, however, is consumed by the reconstruction and does not survive into the result. These uncertainty-aware reconstructions are natural building blocks for what we propose, but on their own, they neither localize reliability by value band nor communicate it, which is found to be fundamental to better understand the evolution of a monitored phenomena.

**Figure 1:**
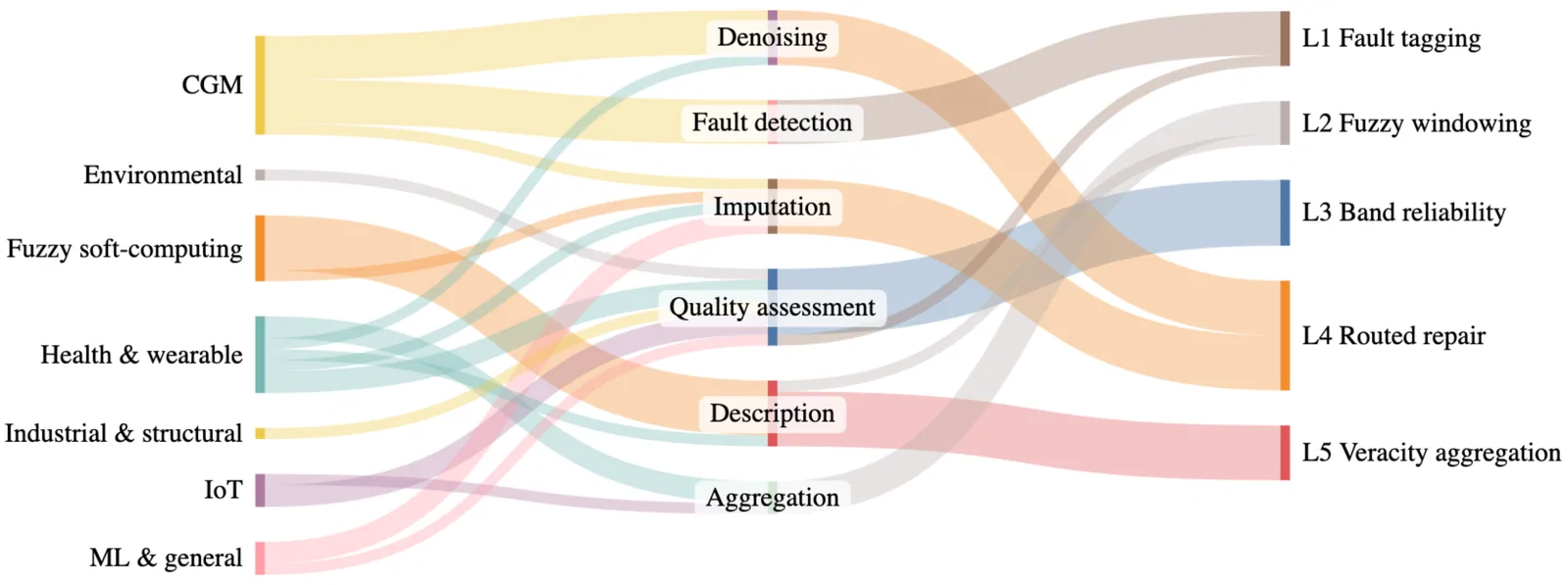
Map of the reviewed literature structuring the domain landscape into sensing application domains, core research objectives, and the layers of the proposed reliability pipeline. Note: All diagrams are original contributions created by the authors specifically for this manuscript.

The means to communicate it come from a further line of work on fuzzy linguistic summarization, in which data is described through protoforms (quantified natural-language statements carrying a degree of truth [33]), subsequently refined with measures of appropriateness and focus [34,35], temporal variants [36] and composite forms [37]. Closest to our own machinery, fuzzy temporal windows and protoforms have been used to summarize health and activity streams in fog and edge settings [38,39,40,41]; in all of it, the subject of the summary is the monitored phenomenon: a trend, a recognized activity or a heart-rate stream. However, we keep the same procedure and turn it on a different subject, the veracity of the data and of the aggregates directly derived from the phenomena. The formal grounding for attaching such a judgment to an aggregate is not provided in temporal or linguistic monitoring, or oriented to sensor monitoring (see Figure 1).

The objective of this work is to characterize the reliability of a sensor stream jointly over the time and value range, and to carry that characterization through to the aggregates derived from the stream, so that each of them is delivered with an explicit degree of veracity. Its novelty lies in combining three elements that, to our knowledge, no existing approach brings together: reliability is computed on the raw signal as a fuzzy quantity conditioned on both time and value band; it routes fault-specific repair whose uncertainty is propagated rather than discarded; and it is reported for every aggregate as a veracity degree and a linguistic protoform. Methodologically, we formulate a five-layer fuzzy pipeline parameterized by a sensor’s range, maximum plausible rate of change and sampling cadence, and instantiate it for CGM with clinical knowledge of glucose dynamics and of sensor failure modes. Fuzzy logic is the natural formalism, because whether a sample is faulty, an interval adequately covered or a value band trustworthy are matters of degree, and forcing them into binary verdicts is what makes conventional quality criteria discard good data and accept bad. For CGM, the expected relevance is clinical: TBR, TIR and TAR drive therapeutic decisions, and knowing which of them can be trusted, and for which periods, is a prerequisite for interpreting them.

To systematically position this work within the current state of the art, we mapped the 30 reviewed studies that inform the proposed pipeline (Table 1). Each study was assigned by the authors to a single application domain (CGM, health & wearable, IoT, environmental, industrial & structural, fuzzy soft-computing, machine learning (ML) & general), to its primary research objective (*Denoising*, Fault detection, hlImputation, Description, Aggregation or Quality assessment) and to the layer of the proposed pipeline it informs (L1 Fault tagging, L2 Fuzzy windowing, L3 Band reliability, L4 Routed repair or L5 Veracity aggregation). Figure 1 visualizes this mapping as a Sankey diagram in which each study flows from its domain to its objective and then to its layer; the width of each flow is proportional to the number of studies on that path, and every flow can be traced to its citations through Table 1. While previous biomedical and industrial monitoring approaches have focused predominantly on sample-level denoising and imputation (feeding L1 and L4), or on abstract data quality frameworks (L3), none of the reviewed studies provides a unified, fuzzy time- and value-band conditioned pipeline that delivers aggregates annotated with linguistic veracity protoforms (L5).

Accordingly, this study aims to develop and evaluate a domain-agnostic framework that models sensor reliability as a fuzzy field over time and value band and propagates it from raw signal data to reliability-aware aggregates, veracity degrees, and linguistic protoforms. The framework is implemented through a five-layer processing pipeline and evaluated on a 14-month real-world CGM recording.

## 2. Materials and Methods

### 2.1. Study Participant and Dataset

The pipeline is evaluated on a long real-world recording [42] from a single adult participant with type 1 diabetes mellitus, monitored under free-living ambulatory conditions. Demographic and clinical characteristics of the participant (such as age, sex, diabetes duration and treatment) are withheld to protect the participant’s privacy, as required by the ethics committee that approved the study (protocol code DIC.21/10.PRY, 2022).

Interstitial glucose was measured with factory-calibrated FreeStyle Libre 3 sensors and retrieved through the architecture described in Section 2.2. For this study, the architecture was configured with a single monitored patient, who authorized a single follower account held by the acquisition tier; the acquisition tier polled the manufacturer’s cloud every 60 s and stored the series as described in Section 2.2.4. The series retrieved for this study contains one reading every five minutes, so it has a nominal cadence of Δ=5 min; the median interval between consecutive stored readings is 6.0 min and 75% of the intervals lie between 4.5 and 6.5 min. Readings are reported in mg/dL; the analyzed data contain 1159 readings at the assay floor (39 mg/dL), 376 below it and 9 above the nominal ceiling of 400 mg/dL, all of which are retained and handled by the saturation primitive (Section 2.4).

The recording spans approximately 14 months (435 calendar days), of which 404 contain at least one reading. Individual days are referred to by their position in the recording (Day 1 being the first recorded day), rather than by calendar date. A day is eligible when it contains at least 120 readings, i.e., 41.7% of the $N_{\Delta }=288$ readings expected at Δ=5 min. The resulting 294 eligible days (58,898 readings) form the dataset analyzed in this work. The threshold is deliberately lower than the 70% active-time criterion of the international consensus [1]. That criterion is a crisp gate that decides whether a 14-day report is valid, whereas the purpose here is to grade incomplete days rather than discard them: coverage enters the analysis continuously through the global veracity of Equation (15), so that days with between 42% and 70% coverage are retained but penalized (159 of the 294 eligible days would also satisfy a 70% criterion). The 120-reading floor only ensures that every day contains enough readings for the fuzzy windows and band memberships to be defined.

### 2.2. Data Acquisition Architecture

**Figure 2:**
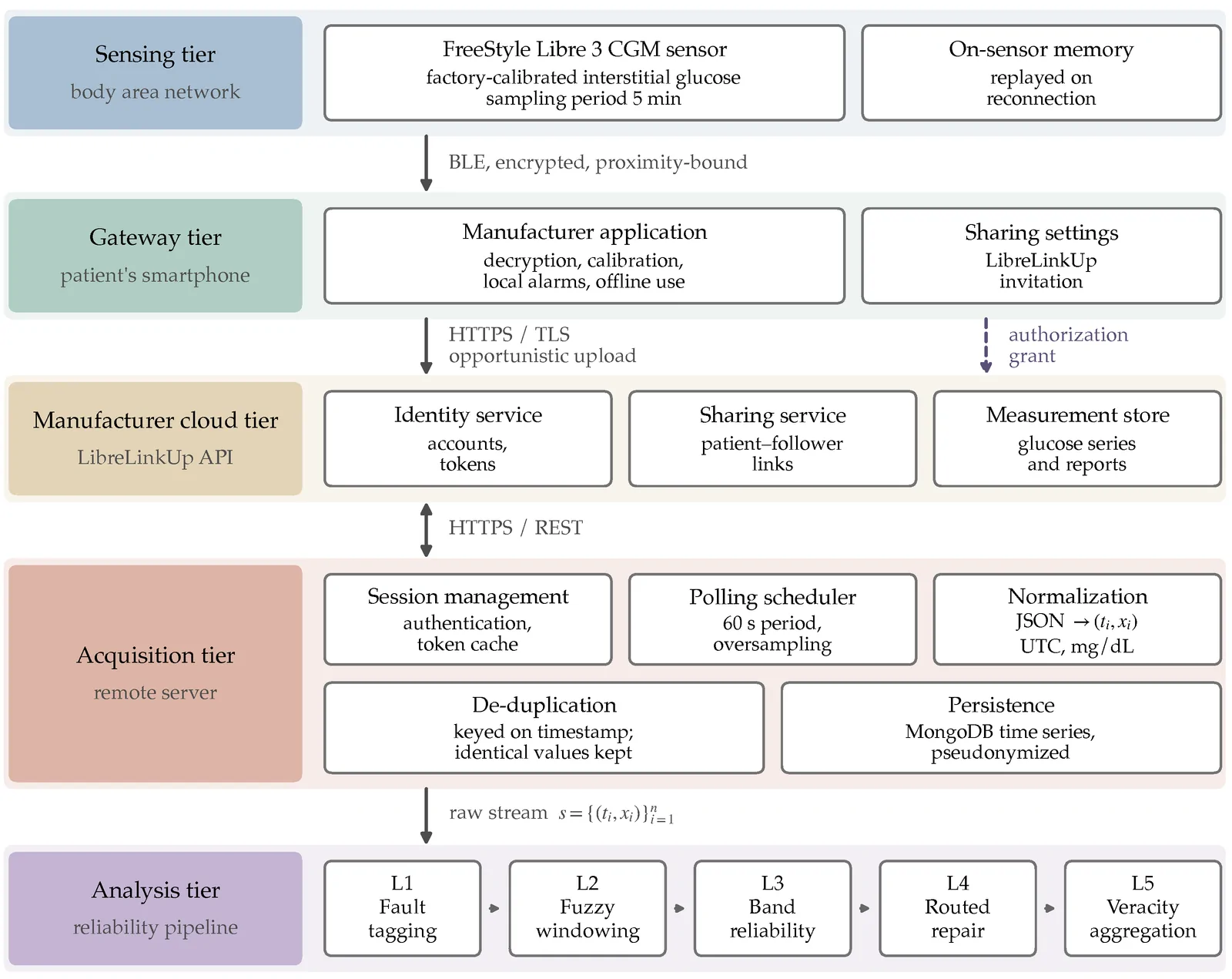
Tiered acquisition architecture for CGM. Solid arrows denote data flow; the dashed arrow is the patient’s authorization grant. The Analysis tier hosts the five layers of the reliability pipeline (L1 fault tagging, L2 fuzzy windowing, L3 band reliability, L4 routed repair and L5 veracity aggregation; Section 2.3). BLE: Bluetooth Low Energy; HTTPS: Hypertext Transfer Protocol Secure; TLS: Transport Layer Security; REST: Representational State Transfer; JSON: JavaScript Object Notation; UTC: Coordinated Universal Time.

This section specifies the architecture through which the glucose stream analyzed in this work is acquired, and delimits the function, the protocol and the configuration parameters of each of its components. It is organized in five tiers (Figure 2):

1. A wearable sensor;
2. A gateway hosted on the patient’s smartphone;
3. A cloud service operated by the device manufacturer;
4. An acquisition tier under institutional control;
5. The analysis tier.

#### 2.2.1. Sensing Tier

The sensing element is a factory-calibrated FreeStyle Libre 3 sensor (Abbott FreeStyle Libre 3: https://www.freestyle.abbott/us-en/products/freestyle-libre-3.html (accessed on 1 July 2026)), which was the device used throughout the recording analyzed in this study (Section 2.1). Like the other members of the FreeStyle Libre family, it measures interstitial glucose through a subcutaneous filament and requires no capillary calibration by the user. The characteristics described below are those of the FreeStyle Libre 3: the sensor has a nominal wear time of 14 days, transmits each reading over an encrypted Bluetooth Low Energy (BLE) link, and the series retrieved for this study contains one reading every five minutes (Δ=5 min; Section 2.1). The link operates over a range of a few meters and requires no network connectivity, so that acquisition continues while the gateway is unreachable. Samples produced during such an interval are retained in the internal memory of the sensor and retransmitted upon reconnection. Acquisition time and arrival time at the gateway are consequently decoupled by an interval bounded by the capacity of that memory.

#### 2.2.2. Gateway Tier

The gateway is the manufacturer’s application executing on the patient’s smartphone. It decrypts the sensor payload, applies the calibration model that converts the raw signal into a glucose concentration, evaluates the alarm thresholds and presents the reading to the patient; none of these functions depends on network availability. The gateway additionally uploads the calibrated series to the manufacturer’s cloud over Hypertext Transfer Protocol Secure (HTTPS). Upload is opportunistic and asynchronous with respect to acquisition: an interval without connectivity postpones transmission rather than discarding samples, so the cloud record of a given interval may be completed after that interval has elapsed. The sharing settings by which the patient authorizes a third party to read the series are configured from this tier.

#### 2.2.3. Manufacturer Cloud Tier

The third tier is operated by the device manufacturer and is reached through the interface that serves its companion application for followers. It provides an identity service that maintains accounts and issues bearer tokens, a sharing service that maintains patient–follower associations, and a measurement store that persists the glucose series and derives the standard clinical reports. Access is region-specific: an authentication request issued against the default endpoint returns, for an account held elsewhere, the region code identifying the endpoint against which the request must be reissued. This tier holds the only authoritative copy of the series; neither the patient nor the acquisition tier retains an independent original, so no reference is available against which a repaired value could be verified.

The authorization model is disjointed from the data plane. The patient issues a sharing invitation to the account under which the acquisition tier authenticates, and the association becomes effective only once that invitation has been accepted, at which point the patient is enumerated among the connections of the account. The grant is revocable, and revocation is not reported as an error condition: the patient ceases to be enumerated.

#### 2.2.4. Acquisition Tier

The acquisition tier is the only component of the architecture under institutional control. It comprises a session manager, a polling scheduler, a normalizer, a de-duplication stage and a persistence store.

Retrieval follows a fixed sequence at every cycle and is mediated by a third-party client library for the manufacturer’s sharing service [43]. The session manager authenticates with the credentials of the authorized account, obtains a bearer token and, when the account is not held in the default region, resolves the regional endpoint against which the request must be reissued. The patients who have authorized that account are then enumerated, which yields the internal identifier of the monitored subject together with the metadata of the active sensor: serial number, activation time, configured target range and alarm thresholds. The reading is finally requested for that identifier. Every request subsequent to authentication carries the bearer token and an account header derived from the user identifier. The interface is the one serving the manufacturer’s companion application rather than a formally specified public application programming interface (API), so its contract is not guaranteed to be stable; the tier is implemented such that a change of contract degrades into an interruption of retrieval rather than into malformed records. Failed requests are not logged as separate events: retrieval is simply reattempted at the next polling cycle, and every record is written by its timestamp, overwriting any previous version of the same reading. Acquisition interruptions therefore appear in the stored series as missing timestamps, indistinguishable from sensor-side gaps, and are handled downstream by the gap primitive and the coverage measures of the pipeline (Section 2.3.1 and Section 2.3.2).

The scheduler polls at a period of 60 s, below the refresh period of the source. The resulting oversampling bounds the latency with which a new reading is observed, which is necessary because the interface exposes no notification channel, and has the consequence that most responses repeat the preceding value. Repetition in the retrieved responses is therefore attributable to the polling policy before it is attributable to the sensor. The de-duplication stage uses the sample timestamp reported by the cloud rather than the value: a reading already stored is discarded when a subsequent poll returns it again, whereas two distinct readings that happen to carry the same value are both retained. Runs of identical values are thus preserved for the stagnation primitive of Section 2.3.1, which would otherwise be unable to fire on any stored series. The normalizer maps each accepted response to a record comprising a Coordinated Universal Time (UTC) timestamp, a value in mg/dL, the trend indicator and the sensor serial number, and the persistence store holds these records as a time series in which the patient identifier is replaced by a study code.

#### 2.2.5. Analysis Tier

The analysis tier receives the stored series as the raw stream *s* and is the only tier that issues a judgment about the data rather than transporting or persisting it. It hosts the reliability pipeline of Section 2.3, organized in five layers (L1–L5).

### 2.3. A Reliability Pipeline for Monitoring

**Figure 3:**
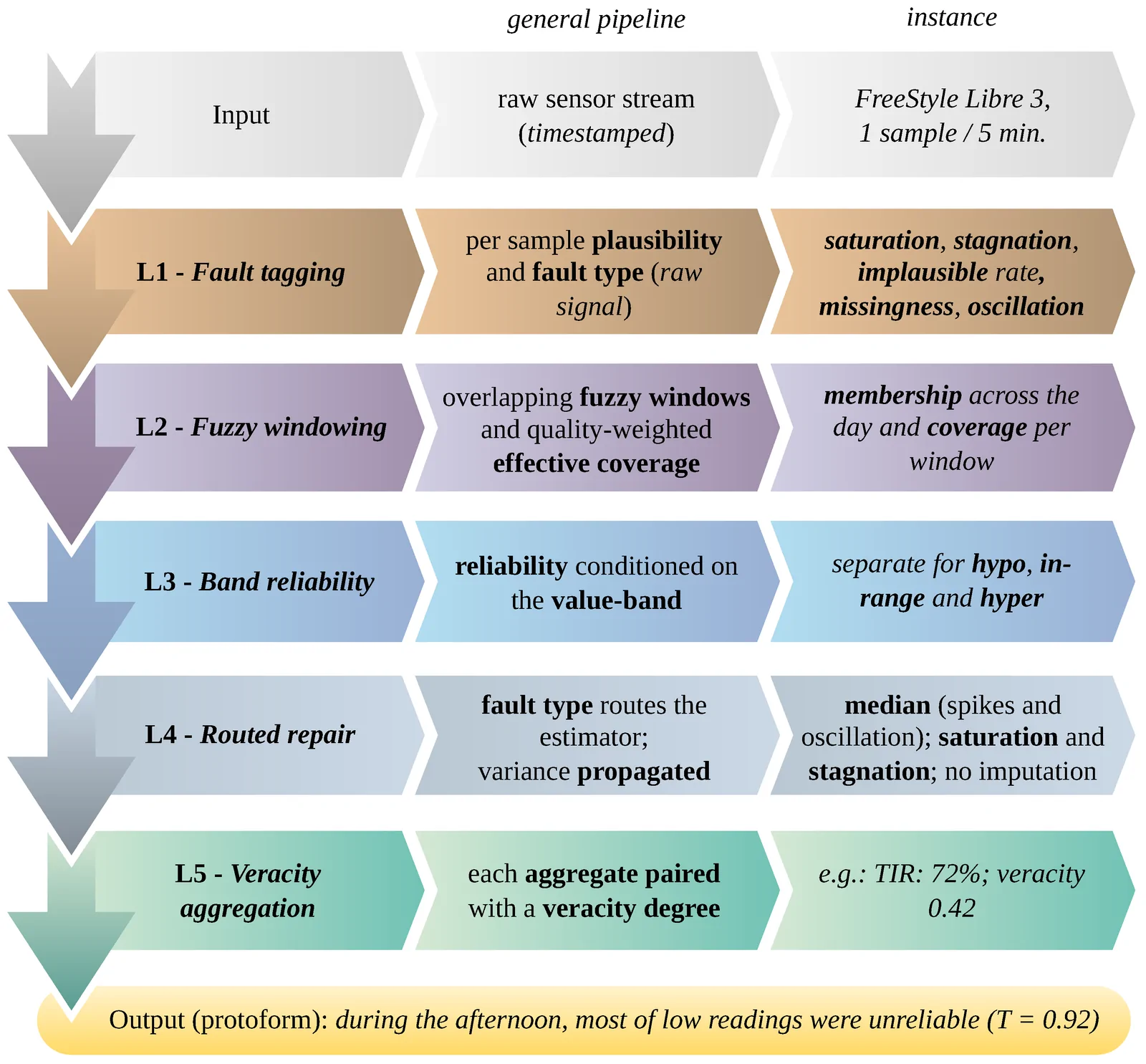
The five-layer reliability pipeline, shown for a generic sensor (left) and instantiated in glucose (right). The left column lists illustrative estimators admitted by each layer; the right column shows the procedures that were actually implemented and evaluated in this study (Section 2.4). The output values are illustrative.

The pipeline turns a raw sensor stream into aggregates that each carry a degree of veracity, together with a linguistic statement of how far they can be trusted. It is organized in five layers, L1–L5 (Figure 3), and is parameterized, for a given sensor, by three quantities: the admissible value range $\left[x_{\min},x_{\max}\right]$, the maximum physiologically plausible rate of change ρ, and the nominal sampling period Δ. Throughout, we write a stream as $s={\left\{\left(t_{i},x_{i}\right)\right\}}_{i=1}^{n}$. A guiding principle keeps apart two concerns that are usually conflated: faults are detected on the raw signal (L1), whereas any repair is a distinct branch (L4) whose every output carries the uncertainty it introduces, so that cleaning can never silently erase the evidence of its own necessity. Every quantity that the layers compute (sample quality, effective coverage, band reliability and veracity) is expressed in [0,1] rather than as a verdict.

#### 2.3.1. L1: Fault Tagging

Fault detection is performed per sample on the raw stream. Each sample is scored by five parameterized primitives, one per failure mode recurrent across monitoring domains, all built from the piecewise-linear ramps

(1) $$R_{a,b}^{↑}\left(z\right)=\min\;\left(1,\max\;\left(0,\frac{z-a}{b-a}\right)\right),\;R_{a,b}^{↓}\left(z\right)=1-R_{a,b}^{↑}\left(z\right),$$

where *a* is the point at which doubt begins and *b* is the point of full membership. For sample *i*, the fault degrees are

(2) $$\begin{array}{cc} \mu_{i}^{\mathrm{sat}} & =\max\;\left(R_{x_{\min},\;x_{\min}+\delta_{\mathrm{lo}}}^{↓}\left(x_{i}\right),\;R_{x_{\max}-\delta_{\mathrm{hi}},\;x_{\max}}^{↑}\left(x_{i}\right)\right), \end{array}$$

(3) $$\begin{array}{cc} \mu_{i}^{\mathrm{rate}} & =R_{\rho ,\;2\rho }^{↑}\left({\dot{x}}_{i}\right),\;{\dot{x}}_{i}=\min\;\left(\frac{|x_{i}-x_{i-1}|}{t_{i}-t_{i-1}},\;\frac{|x_{i+1}-x_{i}|}{t_{i+1}-t_{i}}\right), \end{array}$$

(4) $$\begin{array}{cc} \mu_{i}^{\mathrm{stag}} & =R_{\ell_{a},\;\ell_{b}}^{↑}\left(\ell_{i}\right), \end{array}$$

(5) $$\begin{array}{cc} \mu_{i}^{\mathrm{gap}} & =R_{1.5\Delta ,\;6\Delta }^{↑}\left(g_{i}\right),\;g_{i}=\min\;\left(t_{i}-t_{i-1},\;t_{i+1}-t_{i}\right), \end{array}$$

(6) $$\begin{array}{cc} \mu_{i}^{\mathrm{osc}} & =R_{s_{a},\;s_{b}}^{↑}\left(s_{i}\right),\;s_{i}=\mathrm{sd}\left\{\nabla^{2}x_{j}:\left|j-i\right|\leq 2\right\},\;\nabla^{2}x_{j}=x_{j}-2x_{j-1}+x_{j-2}, \end{array}$$

where $\ell_{i}$ is the length, in samples, of the maximal run of identical consecutive values that contains sample *i*, and boundary samples use a virtual neighbor at distance Δ with the same value. Saturation (Equation (2)) measures proximity to the physical bounds; implausible rate (Equation (3)) compares the rate of change with the slew limit ρ, taking the smaller of the rates to the two neighbors so that an isolated spike scores high while a monotone trend or a single step does not; stagnation (Equation (4)) grows with the length of a run of identical values; missingness (Equation (5)) compares the distance to the nearest neighbor with the expected cadence; and oscillation (Equation (6)) responds to local roughness, measured as the dispersion of second differences. The fault degrees are combined into a per-sample quality through the complement of their probabilistic sum,

(7) $$q_{i}=\prod_{f\in F}\left(1-\mu_{i}^{f}\right),\;F=\left\{\mathrm{sat},\mathrm{rate},\mathrm{stag},\mathrm{gap},\mathrm{osc}\right\},$$

so that several mild faults accumulate rather than being masked by the largest one, and into a dominant fault label,

(8) $$f_{i}=\arg\max_{f\in F}\;\mu_{i}^{f},$$

which routes repair in L4. The saturation, rate and gap primitives are defined only through $\left[x_{\min},x_{\max}\right]$, ρ and Δ; stagnation and oscillation additionally require two shape parameters each ($\ell_{a},\ell_{b}$ and $s_{a},s_{b}$), which must be set for every sensor (Section 2.4).

#### 2.3.2. L2: Fuzzy Windowing

L2 places overlapping windows $W_{j}$, centered at instants $c_{j}$ spaced $h_{c}$ apart, whose membership decays smoothly from the center,

(9) $$\mu_{W_{j}}\left(t\right)=\exp\;\left(-\frac{{\left(t-c_{j}\right)}^{2}}{2s_{W}^{2}}\right),$$

with width $s_{W}$. What a window carries is not the number of samples that fall in it but its quality-weighted effective coverage,

(10) $$c_{W}\;=\;\frac{\sum_{i}\mu_{W}\left(t_{i}\right)\;q_{i}}{\sum_{t\in G_{W}}\mu_{W}\left(t\right)},$$

where $G_{W}$ is the grid of expected sampling instants under Δ over the analyzed period (for one day, the $N_{\Delta }=1440/\Delta$ instants 0,Δ,2Δ,…); the numerator runs over the samples that are actually present. A gap therefore lowers $c_{W}$ continuously instead of producing a discontinuity between neighboring bins, and a sample near a window edge contributes partially to both windows.

#### 2.3.3. L3: Band Reliability

Reliability is resolved not only in time but along the value axis. The range is covered by linguistic bands $\left\{B_{k}\right\}$ with memberships $\mu_{B_{k}}\left(x\right)$. For a window *W* and band $B_{k}$,

(11) $$r_{W,k}\;=\;\frac{\sum_{i}\mu_{W}\left(t_{i}\right)\;\mu_{B_{k}}\left(x_{i}\right)\;q_{i}}{\sum_{i}\mu_{W}\left(t_{i}\right)\;\mu_{B_{k}}\left(x_{i}\right)}$$

weights sample quality by how strongly each reading belongs to the band. A window may thus be reliable in one band and untrustworthy in another: the situation that a single global figure cannot express. When the denominator vanishes (below $10^{-6}$, i.e., no reading of the window belongs to the band), $r_{W,k}$ is undefined and reported as missing.

#### 2.3.4. L4: Routed Repair

Repair is applied selectively, according to the fault label $f_{i}$, and only when the dominant fault degree reaches a routing threshold $\theta_{r}$; samples below it pass through unchanged. Every routed estimator must return a repaired value ${\hat{x}}_{i}$ together with a reconstruction standard deviation $\sigma_{i}$. The routing admits any estimator with this property: for instance, a Hampel or median filter for spikes and oscillations or a Kalman or Gaussian-process smoother for gaps. The estimators actually implemented and evaluated in this study are specified in Section 2.4: a running median for rate and oscillation faults, and flagging without reconstruction for saturation and stagnation, since a sensor pinned at its bound carries no recoverable information; missing stretches are *not* imputed. The reconstruction variance $\sigma_{i}^{2}$ is propagated forward rather than discarded (L5). This branch never feeds back into L1: the fault scores remain those of the raw signal.

#### 2.3.5. L5: Veracity Aggregation

Reconstruction uncertainty enters every aggregate through a reliability weight that attenuates the quality of each sample by the uncertainty of its repaired value,

(12) $$\kappa_{i}=\min\;\left(1,\;\frac{\sigma_{\mathrm{ref}}^{2}+\sigma_{0}^{2}}{\sigma_{\mathrm{ref}}^{2}+\sigma_{i}^{2}}\right),\;\omega_{i}=q_{i}\;\kappa_{i},$$

where $\sigma_{0}$ is the standard deviation assigned to an unrepaired reading, so that $\kappa_{i}=1$ when no repair took place, and $\sigma_{\mathrm{ref}}$ is the scale, in the units of *x*, at which reconstruction uncertainty becomes comparable to the resolution of the value bands. For a band $B_{k}$ and a period containing *n* readings, the *naïve* time in band (TIB) takes every present reading at face value, whereas the *reliability-aware* TIB is computed over the repaired stream and weighted by $\omega_{i}$:

(13) $${\mathrm{TIB}}_{k}^{\mathrm{na}ï\mathrm{ve}}=\frac{1}{n}\sum_{i}\mu_{B_{k}}\left(x_{i}\right),\;{\mathrm{TIB}}_{k}^{\mathrm{aware}}=\frac{\sum_{i}\mu_{B_{k}}\left({\hat{x}}_{i}\right)\;\omega_{i}}{\sum_{i}\omega_{i}}.$$

Each aware estimate is delivered with a band veracity and the profile as a whole with a global veracity,

(14) $$v_{k}=\frac{\sum_{i}\mu_{B_{k}}\left({\hat{x}}_{i}\right)\;\omega_{i}}{\sum_{i}\mu_{B_{k}}\left({\hat{x}}_{i}\right)},$$

(15) $$V=C\cdot \frac{1}{n}\sum_{i}\omega_{i},\;C=\min\;\left(1,\frac{n}{N}\right),$$

where *N* is the number of readings expected in the period under Δ, so that *V* decreases both with low sample reliability and with missing data. The same quantities feed a linguistic summary through a quantifier-driven aggregation in the sense of Kacprzyk et al. [33]. Writing

(16) $$p_{k}=\frac{\sum_{i}\mu_{B_{k}}\left({\hat{x}}_{i}\right)\;\left(1-\omega_{i}\right)}{\sum_{i}\mu_{B_{k}}\left({\hat{x}}_{i}\right)}$$

for the proportion of unreliable readings in band $B_{k}$, the system emits the protoform “during the {P}, $Q^{*}$ of the $\left\{B_{k}\right\}$ readings are unreliable” whenever $p_{k}>\theta_{p}$ and the band contains a minimum mass $\sum_{i}\mu_{B_{k}}\left({\hat{x}}_{i}\right)\geq m_{\min}$. Given a set of fuzzy quantifiers $\left\{Q_{j}\right\}$ defined on the proportion $p_{k}$, the quantifier is the one with the highest truth degree, which is also the truth degree of the following statement:

(17) $$Q^{*}=\arg\max_{j}\;\mu_{Q_{j}}\left(p_{k}\right),\;T=\mu_{Q^{*}}\left(p_{k}\right).$$

The period *P* is the fuzzy period of the day with the highest membership at the unreliability-weighted mean time ${\bar{t}}_{k}=\sum_{i}t_{i}\;\mu_{B_{k}}\left({\hat{x}}_{i}\right)\left(1-\omega_{i}\right)/\sum_{i}\mu_{B_{k}}\left({\hat{x}}_{i}\right)\left(1-\omega_{i}\right)$, and composite protoforms [37] are formed when several such statements must be combined. The output is therefore not a bare number, but an aggregate, its veracity, and a sentence that a practitioner can read.

### 2.4. Instantiation in CGM

The general pipeline is instantiated for CGM by fixing the three sensor parameters and the shape of each fuzzy primitive. We use a nominal cadence Δ=5 min, a value range $\left[x_{\min},x_{\max}\right]=\left[39,400\right]$ mg/dL (the reported floor and ceiling of the assay) and a physiological rate limit derived below. Table 2 lists every parameter of the instantiation together with its basis.

Rate of change (physiological)

The best-justified primitive is the implausible-rate detector. Interstitial glucose cannot change arbitrarily fast: two consecutive readings differ by at most about 10 mg/dL, i.e., a rate of roughly 2 mg/dL·min^−1^ at the 5-min cadence. We therefore let $\mu^{\mathrm{rate}}$ rise from 0 at ρ=2 to 1 at 2ρ=4 mg/dL·min^−1^; expressing the criterion as a rate rather than a fixed per-sample step keeps the bound correct across gaps. A fall from 240 to 100 mg/dL within a single 5-min interval (28 mg/dL·min^−1^), for instance, lies far beyond full membership.

Saturation, stagnation, gap and oscillation

Saturation membership is 1 at or below the assay floor (39 mg/dL) and decays to 0 by 48 mg/dL ($\delta_{\mathrm{lo}}=9$ mg/dL), and rises from 380 to 400 mg/dL at the ceiling ($\delta_{\mathrm{hi}}=20$ mg/dL), so that a reading pinned at, or beyond, a bound is treated as an artifact rather than a true extreme. Stagnation starts at runs of $\ell_{a}=4$ identical readings (20 min) and is full at $\ell_{b}=10$ (50 min): because readings are reported as integers, short repeats are normal during stable periods, and only long runs are suspicious; the de-duplication of the acquisition tier preserves such runs (Section 2.2.4). The gap primitive ramps from 1.5Δ=7.5 to 6Δ=30 min, so a single missed reading is not penalized. Because $g_{i}$ uses the nearest neighbor, it marks readings that are isolated on both sides, as in sparse, intermittent stretches; the absence of readings itself is captured by the effective coverage of Equation (10) and by the coverage factor of Equation (15), not by any sample score. Oscillation ramps from $s_{a}=8$ to $s_{b}=25$ mg/dL of standard deviation of the second differences within five consecutive readings (about 25 min). Unlike the rate limit, these thresholds are not fixed by physiology: they encode expert rules on the failure signatures of CGM (how long a reported value may remain identical, how rough a physiological trace may be), and were set a priori from those rules rather than tuned on the days used for evaluation (Section 2.5).

Value bands

For L3, the glucose axis is covered by three linguistic bands with soft boundaries: hypoglycemic (μ=1 below 65, 0 above 75 mg/dL), in-range, and hyperglycemic (μ=0 below 170, 1 above 190 mg/dL), bracketing the clinical 70–180 mg/dL target range. The in-range membership is $\mu_{\mathrm{in}}=\max\left(0,1-\mu_{\mathrm{hypo}}-\mu_{\mathrm{hyper}}\right)$, so the three memberships sum to one (Equation (20)). The boundaries are centered on the consensus thresholds of 70 and 180 mg/dL [1]; their half-widths of 5 and 10 mg/dL (about 7% and 6% of the threshold) are commensurate with the accuracy of factory-calibrated sensors, so that a reading within measurement error of a clinical threshold belongs partially to both adjacent bands.

Fuzzy windows

L2 uses windows centered every $h_{c}=30$ min from 00:00 to 24:00 (49 windows per day), with width $s_{W}=25$ min. Adjacent windows overlap substantially: each center has membership of 0.49 in its neighbors’ windows.

Routed repair (L4)

Samples whose dominant degree is below $\theta_{r}=0.3$ pass through unchanged. Otherwise, with $m_{i}$, the running median of five consecutive readings centered on *i*, and ${\mathrm{MAD}}_{i}=1.4826\cdot {\mathrm{median}}_{|j-i|\leq 2}\left|x_{j}-m_{j}\right|$, the corresponding median absolute deviation (MAD), the repaired value and its standard deviation (in mg/dL) are

(18) $$\left({\hat{x}}_{i},\;\sigma_{i}\right)=\left\{\begin{array}{cc} \left(x_{i},\;\sigma_{0}\right) & \mathrm{if}\;\max_{f}\mu_{i}^{f}<\theta_{r},\\ \left(m_{i},\;\mu_{i}^{f_{i}}\max({\mathrm{MAD}}_{i},|x_{i}-m_{i}\left|\right)+3\right) & \mathrm{if}\;f_{i}\in \left\{\mathrm{rate},\mathrm{osc}\right\},\\ \left(m_{i},\;50\;\mu_{i}^{f_{i}}+5\right),\;\mathrm{flagged} & \mathrm{if}\;f_{i}\in \left\{\mathrm{sat},\mathrm{stag}\right\},\\ \left(x_{i},\;20\;\mu_{i}^{f_{i}}+3\right) & \mathrm{if}\;f_{i}=\mathrm{gap}, \end{array}\right.$$

with $\sigma_{0}=2$ mg/dL. Readings dominated by the implausible-rate or oscillation memberships are thus replaced by a local robust estimate whose uncertainty grows with both the local scatter and the fault degree. Readings pinned at the assay floor or stuck at a constant value are *flagged and left unreconstructed*: the median is a mere placeholder and its standard deviation of up to 55 mg/dL spans more than one band, so that such readings carry essentially no band information ($\kappa_{i}\approx 0.03$ in Equation (12)). Readings isolated by gaps keep their value with an inflated uncertainty. No value is generated for missing timestamps: missing stretches are not imputed or interpolated, and enter the analysis only through $c_{W}$ and the coverage factor *C*.

Veracity aggregation and protoforms (L5)

The clinical profile is the triple TBR, TIR and TAR: that is, the TIB of each glucose band, computed with Equation (13) over each day. We set $\sigma_{\mathrm{ref}}=10$ mg/dL, the width of the transition of the hypoglycemic band, so that a reconstruction uncertainty comparable to that width roughly halves the weight of a reading ($\kappa_{i}=0.52$ at $\sigma_{i}=10$ mg/dL), and N=288 readings per day in Equation (15). Protoforms are emitted for $p_{k}>\theta_{p}=0.35$ with $m_{\min}=3$, using the quantifiers and periods of the day of Equations (21) and (22); for instance, the system states *“during the afternoon, almost all of the low readings are unreliable”*, with truth degree $T=\mu_{\mathrm{almost}\;\mathrm{all}}\left(p_{\mathrm{low}}\right)$.

Linguistic terms

All linguistic terms of L3 and L5 are trapezoidal fuzzy sets,

(19) $${\mathrm{Tr}}_{a,b,c,d}\left(z\right)=\max\;\left(0,\;\min\;\left(\frac{z-a}{b-a},\;1,\;\frac{d-z}{d-c}\right)\right),$$

where a side with a=b (or c=d) is taken as constant and equal to one, so that ramps are obtained as particular cases. The glucose bands (in mg/dL), the periods of the day (in hours) and the quantifiers (on the proportion $p_{k}$) are

(20) $$\begin{array}{cc} \; & \mu_{\mathrm{hypo}}={\mathrm{Tr}}_{0,0,65,75},\;\mu_{\mathrm{hyper}}={\mathrm{Tr}}_{170,190,x_{\max},x_{\max}},\;\mu_{\mathrm{in}}=\max\;\left(0,1-\mu_{\mathrm{hypo}}-\mu_{\mathrm{hyper}}\right),\\ \; & \mu_{\mathrm{night}}={\mathrm{Tr}}_{0,0,5.5,6.5},\;\mu_{\mathrm{morning}}={\mathrm{Tr}}_{5.5,6.5,11.5,12.5}, \end{array}$$

(21) $$\begin{array}{cc} \; & \mu_{\mathrm{afternoon}}={\mathrm{Tr}}_{11.5,12.5,17.5,18.5},\;\mu_{\mathrm{evening}}={\mathrm{Tr}}_{17.5,18.5,24,24}, \end{array}$$

(22) $$\begin{array}{cc} \; & \mu_{\mathrm{about}\;\mathrm{half}}={\mathrm{Tr}}_{0.30,0.40,0.50,0.65},\;\mu_{\mathrm{most}}={\mathrm{Tr}}_{0.50,0.65,0.80,0.90},\;\mu_{\mathrm{almost}\;\mathrm{all}}={\mathrm{Tr}}_{0.80,0.90,1,1}, \end{array}$$

The three families are fuzzy partitions: the memberships of adjacent terms sum to one in every transition. For the periods, the transitions last one hour and are centered at 06:00, 12:00 and 18:00. For the quantifiers, the partition guarantees that the selected quantifier of Equation (17) always has a truth degree of at least 0.5 for $p_{k}>\theta_{p}$.

### 2.5. Evaluation Procedure

Reference days

Ten days of the recording were selected as reference cases on the basis of expert knowledge of CGM failure modes, informed by feedback from the participant and from the participant’s physician on the incidents and anomalous readings observed during the recording: two clean control days and eight days exhibiting characteristic faults (missing stretches, anomalous readings in the low or high band, oscillation, and clamping at the assay floor), each labeled with its dominant fault character (Table 3). Two of the artifact days (Days 90 and 184) are dominated by missing data and contain fewer than 120 readings, so they fall outside the eligible set; the remaining six are marked in the phenotyping analysis (Section 2.6). Because these labels and the fault rules of L1 derive from the same body of clinical knowledge, agreement on the reference days is a consistency check of the implementation rather than an independent validation of the detector. Nevertheless, the participant and the physician provided their feedback without access to the pipeline outputs: the aim of the method is to model their knowledge of when the data can be trusted, not to adjust that knowledge to the method, so that no “false” knowledge introduced by imputation or smoothing could leak into the reference.

Naïve versus reliability-aware profiles

On the reference days, the profile obtained by taking every reading at face value (the naïve estimate of Equation (13)) is contrasted with the reliability-aware profile of four quantities: the three times in band and the global veracity *V*.

Sensitivity to the reconstruction uncertainty

To isolate the contribution of the reconstruction uncertainty propagated from L4, every reliability-aware analysis (reference days and phenotype clustering) is repeated with $\kappa_{i}=1$ in Equation (12), i.e., weighting each reading by its quality $q_{i}$ alone.

### 2.6. Phenotype Clustering

To assess whether reliability-aware processing changes how a patient is characterized over time, or only on the odd corrupted day, we profiled every eligible day of the recording (Section 2.1) and clustered the days in the time-in-band space, once from the naïve profiles and once from the reliability-aware ones. For each day, the features are the times below and above range (TBR, TAR) of Equation (13), standardized to zero mean and unit variance across days; TIR is omitted because TIR=100−TBR−TAR carries no additional information. Each set of profiles is partitioned with *k*-means (Euclidean distance, 10 random initializations, random seed 0; scikit-learn). The number of clusters was chosen among k=2,…,8 by two criteria evaluated on both the naïve and the reliability-aware profiles: the mean silhouette coefficient, and the stability of the partition, measured as the mean adjusted Rand index (ARI) between the full-data partition and 100 partitions fitted on random subsamples of 80% of the days. k=3 maximized both criteria (naïve profiles: silhouette 0.46, ARI 0.97; aware profiles of the profilable days: silhouette 0.49, ARI 0.99, with 5th-percentile ARI above 0.94 in both cases). The two partitions are fitted independently: the reliability-aware clustering is a separately trained model, not a relabeling of the naïve one. Consistently with the meaning of the global veracity, days with V<0.5 (Equation (15)) are considered to be too unreliable to profile and receive no reliability-aware phenotype: the aware partition is fitted only on the remaining days, with *k* re-selected on them by the same criteria, whereas the naïve partition, which ignores reliability, assigns a phenotype to every eligible day. Each day is therefore classified as keeping its naïve phenotype, changing it, or becoming non-profilable. To compare the two partitions, clusters are matched one-to-one by maximizing their overlap (Hungarian algorithm on the k×k contingency table), using the profilable days, and a profilable day is said to change phenotype when its matched labels differ. To compare the veracity-based exclusion with the crisp consensus rule, the profilable days are also contrasted with the days that meet the 70% active-time criterion [1].

## 3. Results

### 3.1. Per-Sample Quality on the Studied Days

With the memberships of Table 2, the clean control days retain a mean quality of 0.98–0.99, with 2.5% of their readings below q=0.5 at most, whereas every artifact day drops to 0.51–0.87, with 13–49% of its readings below 0.5 (Table 3). The fault rules thus mark the days that the participant and the physician identified as problematic while leaving the clean days almost untouched, which confirms that the knowledge encoded in L1 is implemented consistently (Section 2.5).

### 3.2. Fault Detection on Representative Days

**Figure 4:**
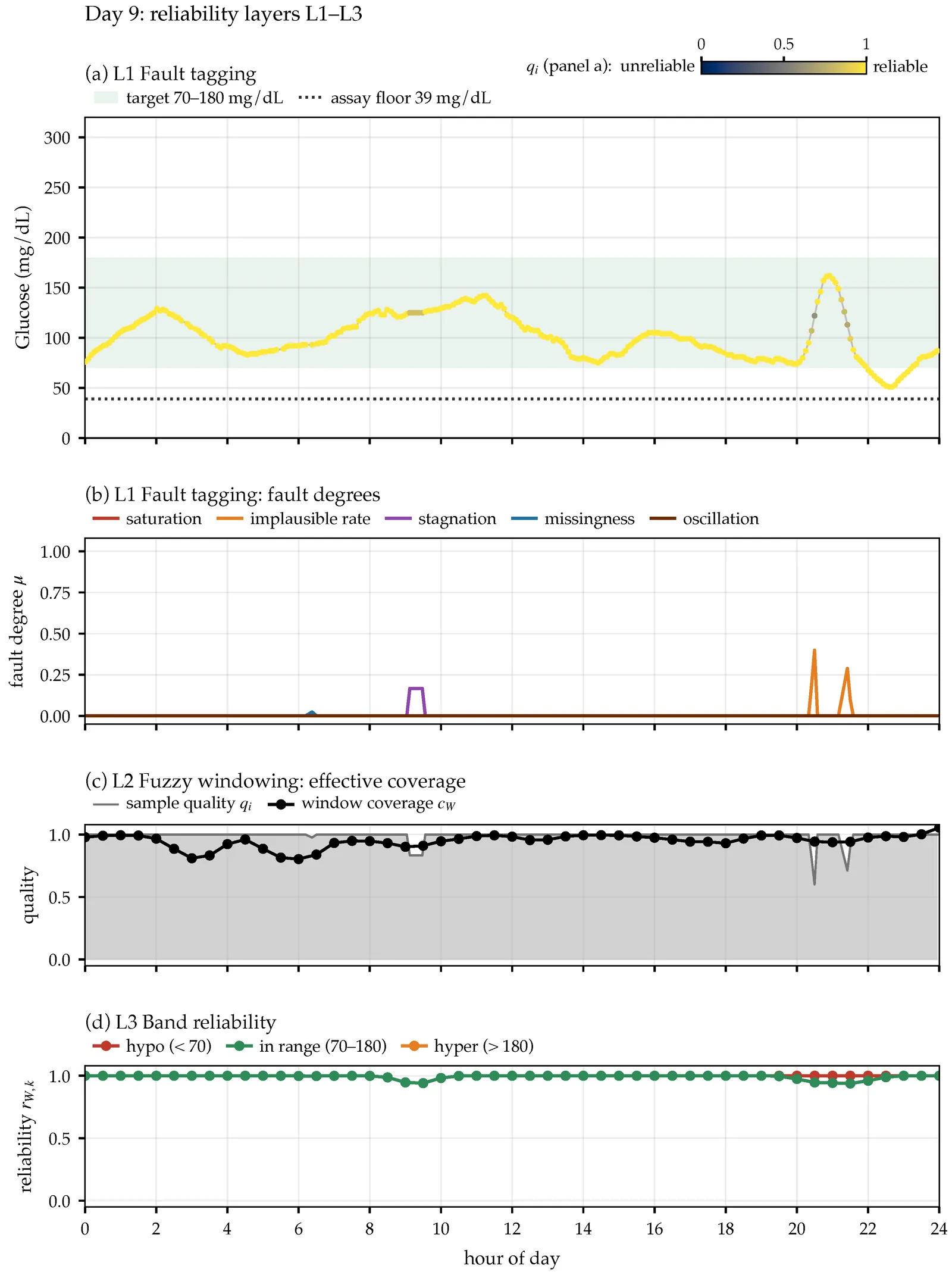
Reliability layers L1–L3 on a *clean control* day (Day 9 of the recording). (**a**) L1 fault tagging: each glucose reading coloured by its quality $q_{i}$ (the colour bar applies to this panel only: 0 = unreliable, 1 = reliable); the shaded band marks the 70–180 mg/dL target range, the dotted line the assay floor (39 mg/dL), and hatched areas intervals without readings for more than 30 min. (**b**) Fault degree $\mu_{i}^{f}$ of each of the five primitives (Equations (2)–(6)). (**c**) L2 fuzzy windowing: sample quality $q_{i}$ (grey area) and quality-weighted effective coverage $c_{W}$ of each window (black dots; Equation (10)). (**d**) L3 band reliability $r_{W,k}$ of the hypoglycemic, in-range and hyperglycemic bands (Equation (11)); gaps mark windows in which no reading belongs to the band. Quality and coverage stay near one all day.

**Figure 5:**
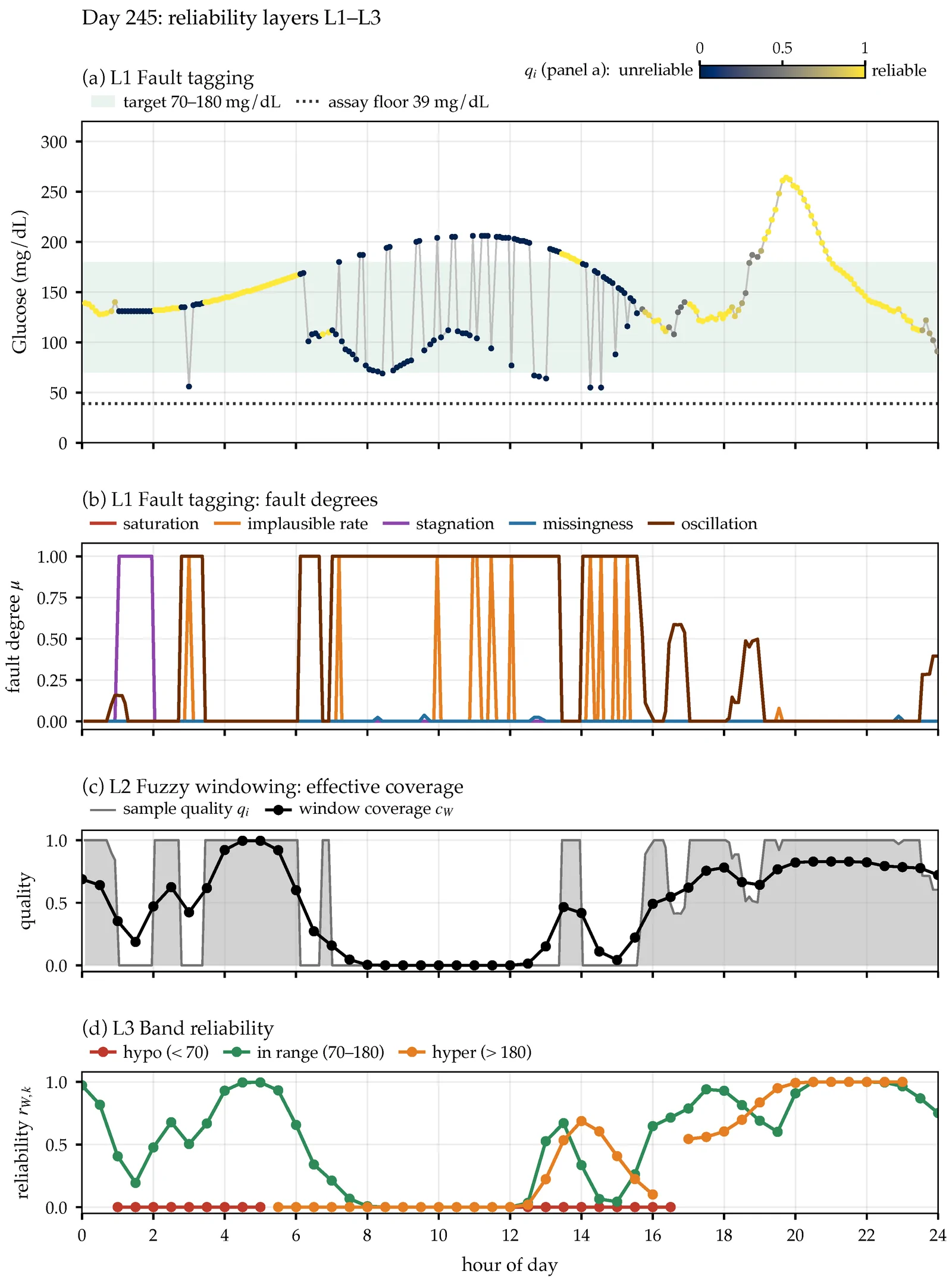
Reliability layers L1–L3 on a day of physiologically impossible *oscillation* (Day 245), with the layout of Figure 4. Panels: (**a**) readings coloured by $q_{i}$ (colour bar for panel (**a**) only); (**b**) fault degrees; (**c**) $q_{i}$ (grey area) and window coverage $c_{W}$ (dots); (**d**) band reliability $r_{W,k}$; hatched areas, no readings for more than 30 min. The oscillation between about 6 and 16 h drives quality, coverage and every band reliability down, whereas the calm intervals stay reliable.

**Figure 6:**
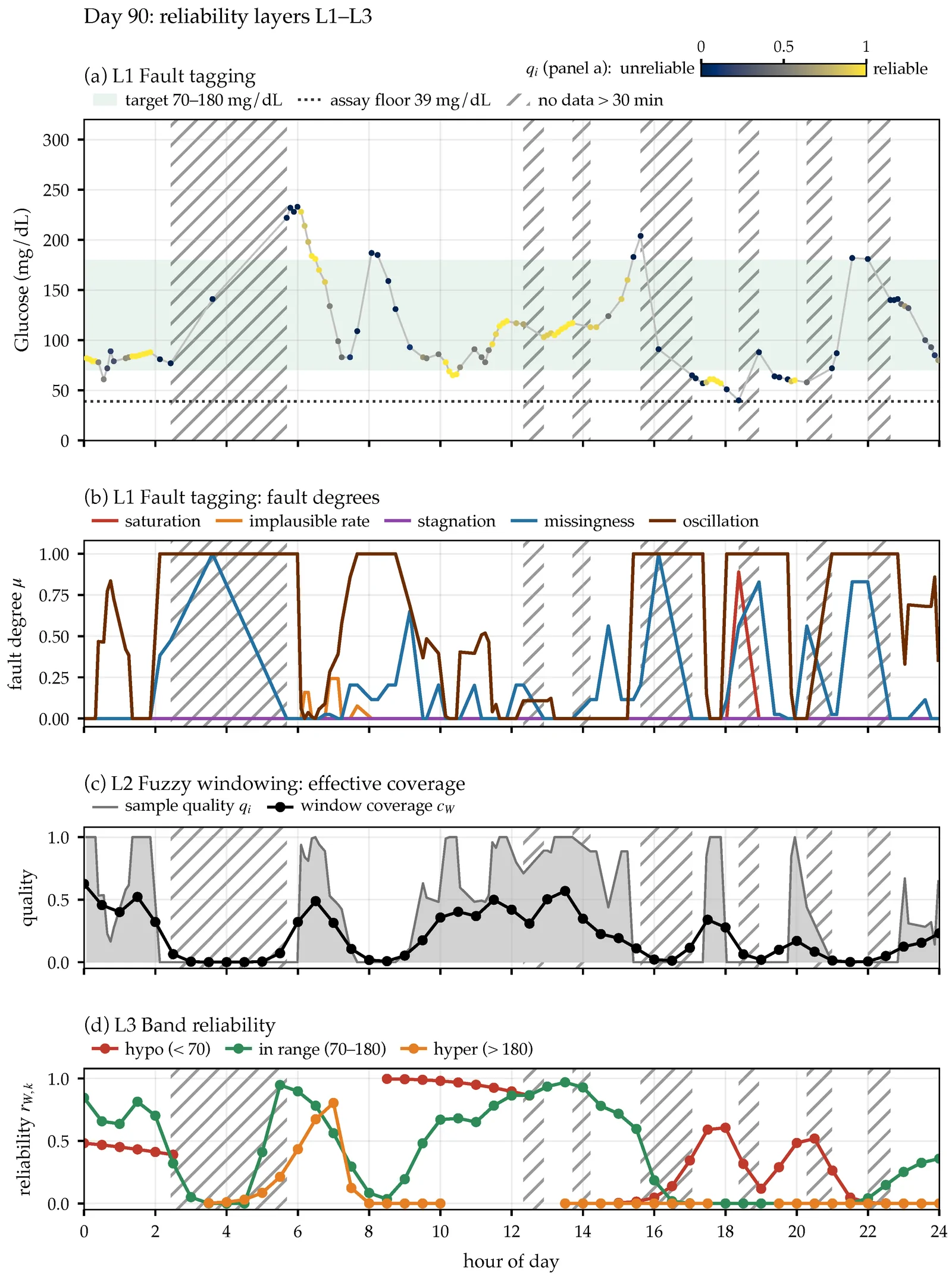
Reliability layers L1–L3 on a day with long *missing* stretches (Day 90), with the layout of Figure 4. Panels: (**a**) readings coloured by $q_{i}$ (colour bar for panel (**a**) only); (**b**) fault degrees; (**c**) $q_{i}$ (grey area) and window coverage $c_{W}$ (dots); (**d**) band reliability $r_{W,k}$; hatched areas, no readings for more than 30 min. Effective coverage falls where the sensor is absent, so the aggregates are marked incomplete rather than interpolated.

Figure 4, Figure 5 and Figure 6 run the instantiated pipeline (L1–L3) on three contrasting days. On a clean control day (Day 9; Figure 4), quality stays at q≈0.99, effective coverage sits near one, and every band reliability is close to unity: the method is silent when there is nothing to flag. On a day dominated by physiologically impossible oscillation (Day 245; Figure 5), with the sensor alternating between roughly 90 and 200 mg/dL within minutes, the implausible-rate and oscillation memberships fire across the affected interval, the effective coverage $c_{W}$ collapses towards zero there, and the in-range, hypoglycemic and hyperglycemic assessments are all marked untrustworthy for that stretch, while the calm morning and late evening remain reliable. On a day with long missing stretches (Day 90; Figure 6), the gap membership dominates instead: effective coverage falls wherever the sensor is absent and the band reliabilities are low or undefined there, so the day’s aggregates are honestly reported as incomplete rather than silently interpolated.

### 3.3. Repair and Reliability-Aware Profiling of the Most Corrupted Day

**Figure 7:**
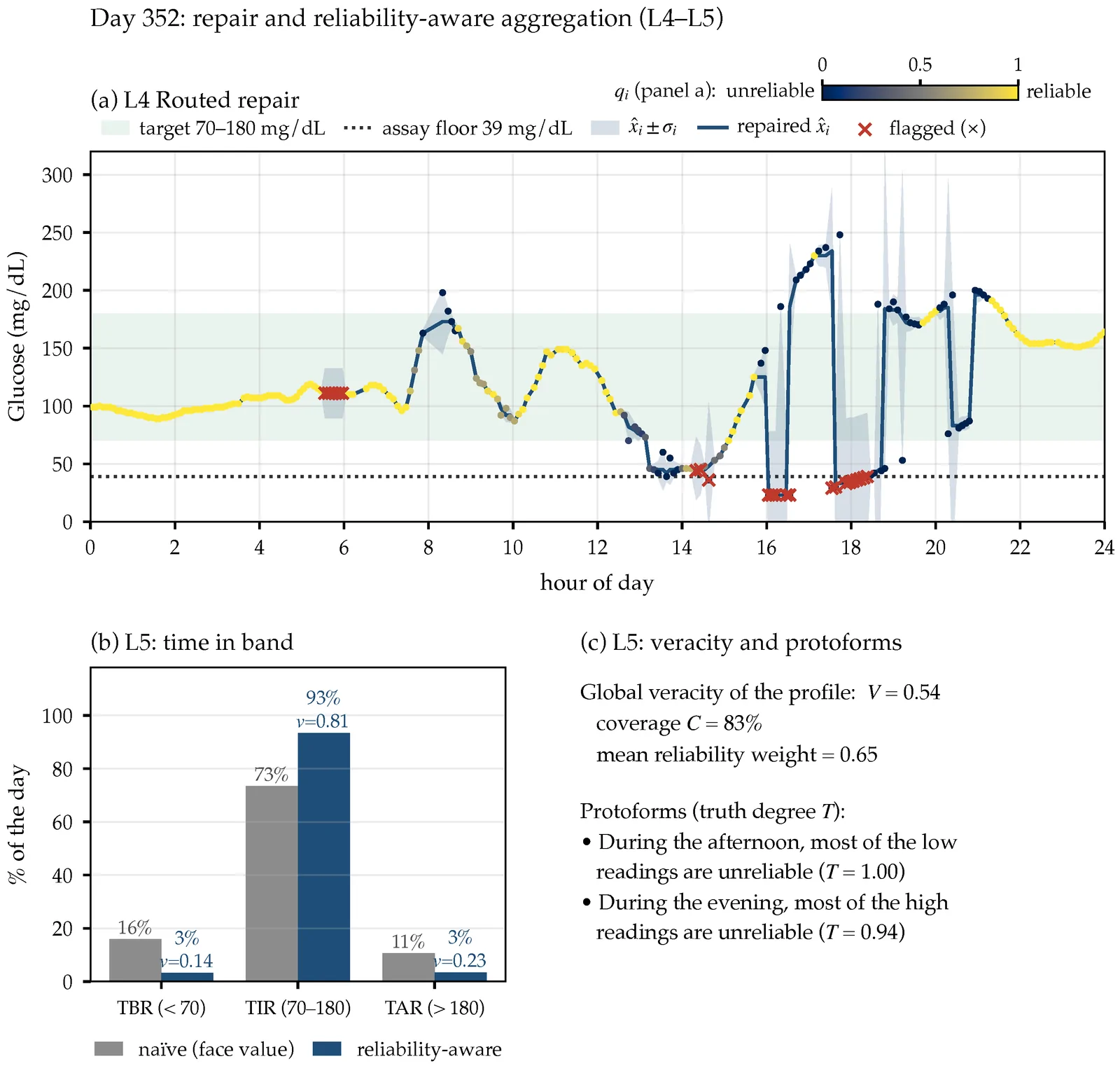
Repair and reliability-aware aggregation (L4–L5) on the most corrupted reference day (Day 352). (**a**) L4 routed repair: raw readings coloured by their quality $q_{i}$ (the colour bar applies to this panel only), repaired values ${\hat{x}}_{i}$ (line) with their $\pm \sigma_{i}$ reconstruction band (shaded), and readings at the assay floor or stuck at a constant value, flagged (×) (Equation (18)); the green band marks the 70–180 mg/dL target range and the dotted line the assay floor. (**b**) TBR, TIR and TAR from the naïve profile, which takes every reading at face value (grey), and from the reliability-aware profile (blue), annotated with the band veracity $v_{k}$ (Equations (13) and (14)). (**c**) Global veracity *V* (Equation (15)), with the coverage *C* and the mean reliability weight, and the protoforms emitted with their truth degrees (Equation (17)).

Figure 7 illustrates the performance of L4 and L5 on Day 352, selected as the most corrupted day according to the multi-fault compound criterion. Although some days in Table 3 exhibit a lower global mean quality score ($\bar{q}$), mainly due to extended missing gaps or isolated noise spikes, Day 352 uniquely combines persistent assay floor-clamping (saturation at ≤39 mg/dL) with high-amplitude physiological oscillations (>100 mg/dL within minutes). This combination produces a substantial distortion in the estimated TBR: the naïve profile reports ${\mathrm{TBR}}_{\mathrm{naive}}=16\%$, whereas the reliability-aware profile reports ${\mathrm{TBR}}_{\mathrm{aware}}=3\%$, a reduction of about 80%. More importantly, the reliability-aware profile assigns a veracity of only 0.14 to the low-range estimate and generates the protoforms *“during the afternoon, almost all of the low readings are unreliable”* (T=0.63) and *“during the evening, most of the high readings are unreliable”* (T=1.00). In contrast, on a clean control day (Day 9), the two profiles remain essentially unchanged (5% versus 6% below range), with all band veracities close to one. Thus, the method remains effectively silent when the underlying data do not provide evidence of relevant quality problems.

### 3.4. Naïve Versus Reliability-Aware Profiles

**Figure 8:**
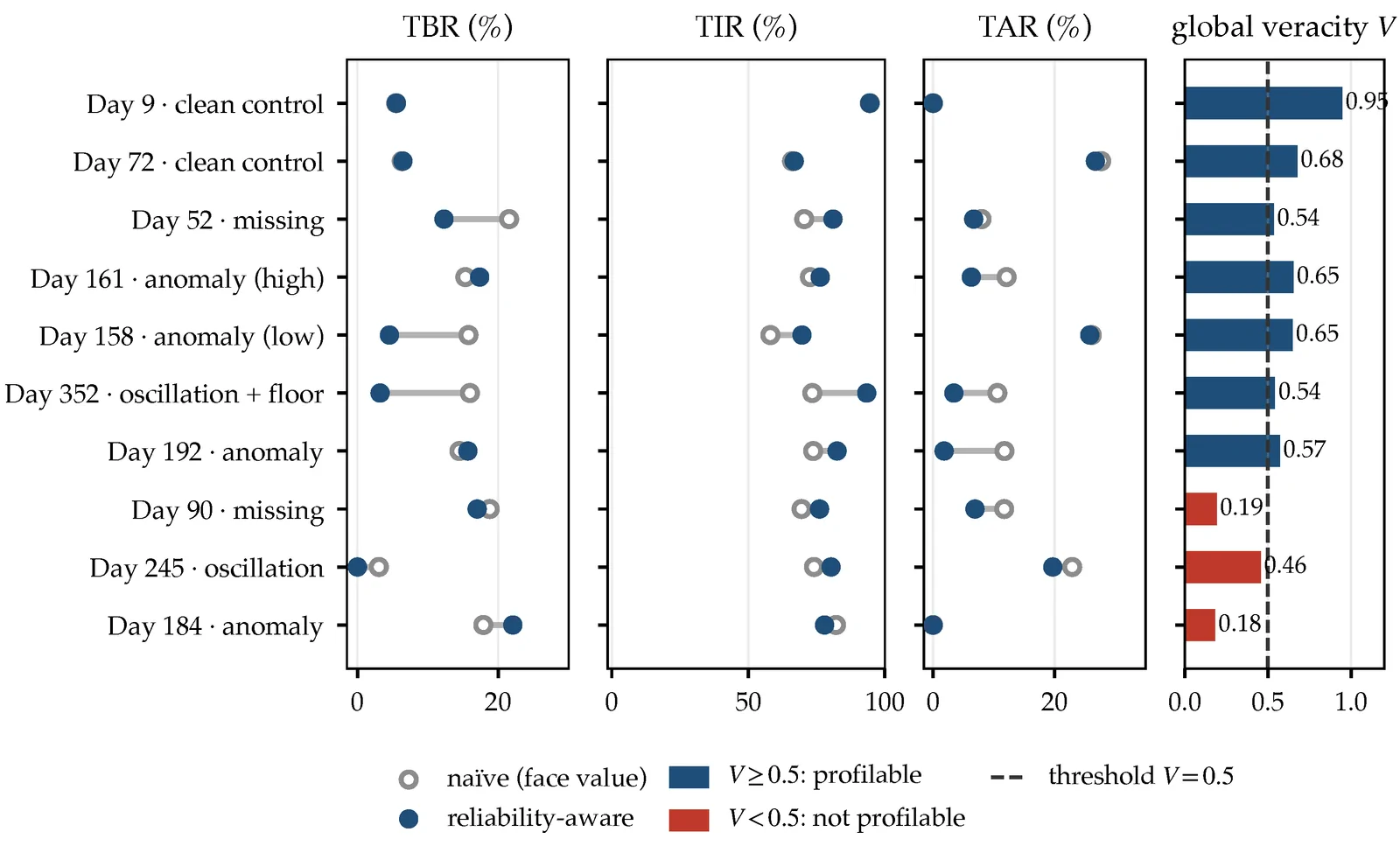
Naïve versus reliability-aware profiles of the ten reference days (Table 3). Rows: day of the recording and its fault character. First three panels: TBR, TIR, TAR, Equation (13), each on its own percentage scale; open grey circles, naïve profile (every reading at face value); filled blue circles, reliability-aware profile; the grey segment joins the two estimates of the same day. Right panel: global veracity *V* of the reliability-aware profile (Equation (15)); days below the dashed threshold V=0.5 (red) are considered too unreliable to profile.

Figure 8 contrasts the naïve and reliability-aware profiles of the ten reference days, with each metric on its own scale. On the clean control days, the two profiles coincide and the global veracity is high: there is nothing to correct and the method says so. On the corrupted days, the profiles separate, chiefly in TBR and TAR, where floor-clamped, oscillating or anomalous readings are discounted, and the global veracity drops; on three of them (Days 90, 245 and 184), it falls below V=0.5 so that no profile would be drawn at all. On Day 352, a naïve TBR of 16% (enough to prompt a change of therapy) shrinks to 3%, and the low-range estimate carries a veracity of 0.14. Disregarding the reconstruction uncertainty ($\kappa_{i}=1$; Section 2.5) changes these profiles by at most 1.4 percentage points and the global veracity by at most 0.02.

Because the profile veracity folds the day’s coverage into its quality (it is the mean quality scaled by the fraction of the day that is actually recorded), it also flags days that are clean but sparsely worn: the second control day (Day 72), recorded for only 69% of its hours, scores a veracity of 0.68 despite pristine samples.

### 3.5. Impact on Phenotyping over the Whole Recording

**Figure 9:**
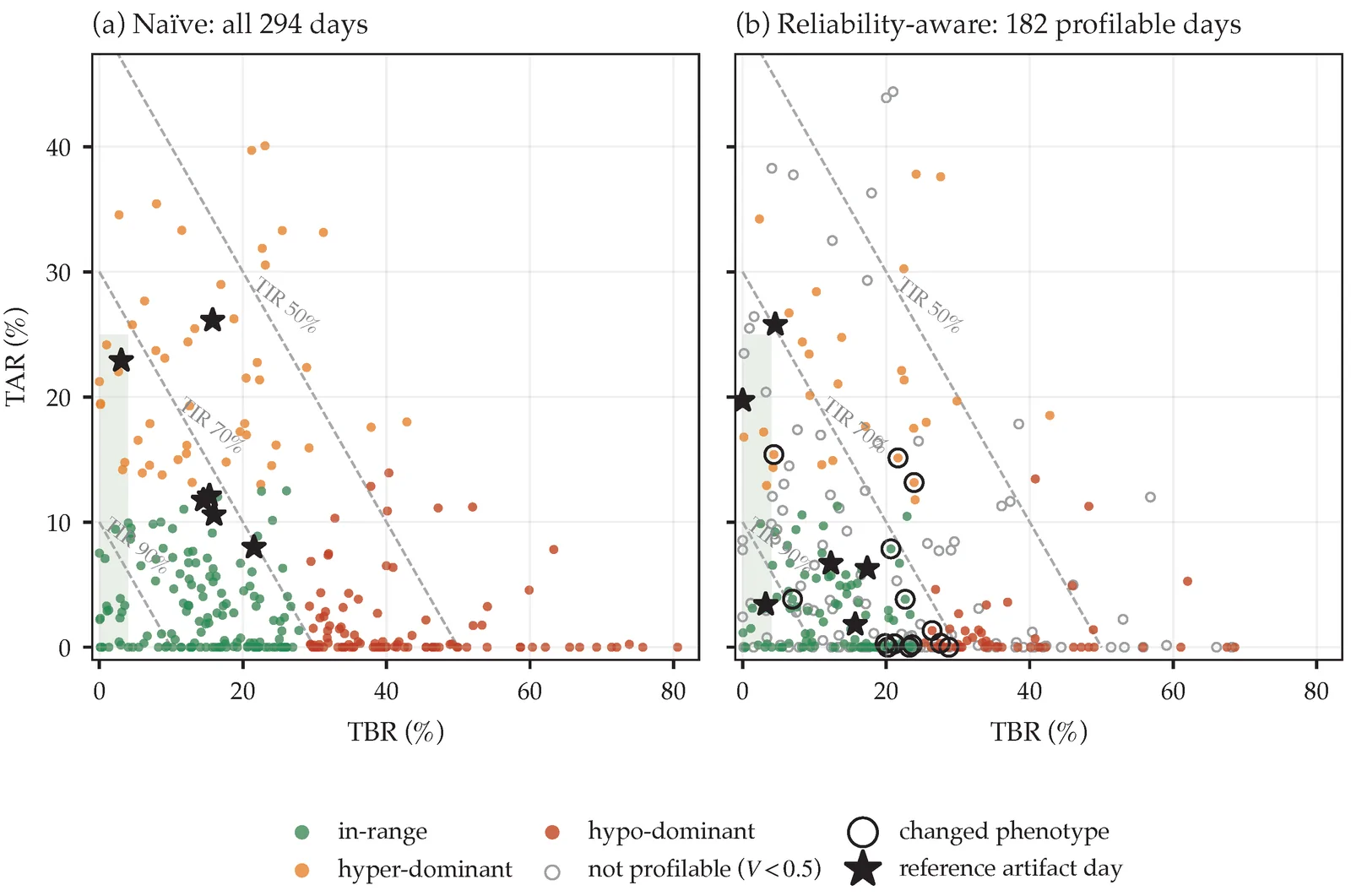
Daily glycemic phenotypes over the 294 eligible days, in the plane of TBR and TAR; TIR is implicit (dashed iso-TIR lines), and the shaded corner marks the consensus targets TBR < 4% and TAR < 25%. Colours: phenotypes obtained by *k*-means (k=3) and matched between panels (Section 2.6); the number of days in each phenotype is given in Section 3.5. (**a**) Naïve profiles: every eligible day is phenotyped. (**b**) Reliability-aware profiles: days with global veracity V<0.5 (open grey circles) receive no phenotype; black rings mark profilable days whose phenotype changed with respect to (**a**). Stars: reference artifact days (Table 3).

Figure 9 shows the daily phenotypes over the whole recording (Section 2.6). The naïve profiles separate into three phenotypes: an in-range phenotype (145 days; mean TBR 14.5%, TIR 82.2%, TAR 3.3%), a hyper-dominant one (50 days; 14.7%, 63.1%, 22.1%) and a hypo-dominant one (99 days; 42.3%, 56.0%, 1.7%). Once reliability is taken into account, 112 of the 294 days (38%) have V<0.5 and receive no phenotype; the remaining 182 days again form three phenotypes with similar centroids (in-range: 99 days, 13.0%, 84.4%, 2.6%; hyper-dominant: 30 days, 14.8%, 64.0%, 21.2%; hypo-dominant: 53 days, 37.4%, 61.5%, 1.2%).

Of the 294 days, 167 (57%) keep their naïve phenotype, 15 (5%) change it and 112 (38%) become non-profilable, so that 43% of the days receive a different characterization than in the naïve analysis. Phenotype changes occur at the boundary between the in-range and hypo-dominant phenotypes (6 days leave the in-range phenotype and 9 the hypo-dominant one), whereas non-profilable days come from all three phenotypes (34%, 46% and 40% of the in-range, hyper-dominant and hypo-dominant days, respectively). Of the six eligible reference artifact days, five keep their phenotype and one (Day 245) becomes non-profilable. Of the 112 non-profilable days, 60 would be excluded by their coverage alone (C<0.5), whereas 10 meet the 70% active-time criterion of the consensus [1] but have low sample reliability; conversely, 33 profilable days have between 42% and 70% coverage and would be discarded by that criterion. Disregarding the reconstruction uncertainty ($\kappa_{i}=1$) yields 174, 16 and 104 days that keep, change and lose their phenotype, respectively.

## 4. Discussion

### 4.1. Principal Findings

The results show that reliability-aware processing alters the knowledge derived from CGM at two scales. At the scale of a single day, it leaves clean days essentially untouched (Days 9 and 72; Figure 8) while correcting metrics distorted by artifacts: on Day 352, a naïve TBR of 16% falls to 3% once floor-clamped and oscillating readings are discounted, and the low-range estimate is explicitly marked as untrustworthy (v=0.14). At the scale of the whole recording, the main effect is not that days change phenotype (only 5% do, all at the boundary between the in-range and hypo-dominant phenotypes) but that 38% of the eligible days carry too little reliable information to be phenotyped at all; in total, 43% of the days receive a different characterization from that of the naïve analysis. The phenotypes themselves are stable, since their centroids barely move once unreliable days are excluded: the patient’s glycemic profile is not an artifact of corrupted data, but a substantial part of the evidence upon which a naïve analysis rests is.

### 4.2. Graded Versus Crisp Sufficiency Criteria

The global veracity behaves as a graded counterpart of the 70% active-time criterion of the international consensus [1]. The two criteria largely agree, but they disagree in an informative way: 33 days with 42–70% coverage but reliable readings would be discarded by the crisp rule and are retained by the veracity, whereas 10 days that satisfy the rule are excluded because their readings are unreliable. Coverage alone, which is what the consensus assesses, is therefore neither sufficient nor necessary to decide whether a day can be interpreted: the quality of what was recorded matters as much as how much was recorded.

### 4.3. Comparison with CGM Signal Processing

These results are consistent with, and extend, previous work on CGM data processing. Denoising approaches, from adaptive Kalman filtering [11] and smart-sensor cascades [2,10] to the Bayesian treatment of coloured noise [12], improve the signal but return a cleaned series without stating how much of it was inferred; deep imputation of corrupted stretches [13] likewise reconstructs values without carrying their uncertainty into the clinical metrics. Fault-detection methods for CGM, based on statistical criteria [15], model-based detection for closed-loop control [16], clustering [17] or explicit models of disconnections and compression [14], share our premise that faults must be identified on the raw signal, but they produce sample-level flags aimed at real-time use. The present pipeline differs in what it delivers rather than in its ability to detect a given fault: graded fault degrees are carried forward to the aggregates that clinicians actually read, so that the effect of a fault on TBR, TIR or TAR is quantified and stated (Figure 7). The observation that a stretch of floor-clamped readings can inflate the TBR of a day several-fold also complements reports that CGM-derived metrics depend on the sensor and carry a systematic bias [3,4]: besides device-level bias, day-level faults distort the same metrics, and they can be detected from the data themselves.

### 4.4. Comparison with Data-Quality and Uncertainty-Aware Approaches

Outside glucose, signal-quality indices for wearable photoplethysmography grade each segment and route the subsequent processing [18,19], an idea close to our fault-routed repair; data-quality frameworks for the IoT, wearables and industrial monitoring attach reliability flags to the stream [21,22,23,25]; and uncertainty-aware imputation returns a variance with each reconstructed value [29,30,31]. In these approaches, however, quality is a scalar per segment or per stream, and the uncertainty is consumed by the reconstruction, as with the reliability index of Hosseini and Moeini [32], rather than conditioned on the value range and attached to the result. The band-conditioned veracity addresses precisely this gap: Days 158 and 352 are reliable in range but unreliable in the low band (Figure 7), a situation that a single quality score per day cannot express. As for the propagated reconstruction uncertainty, the sensitivity analysis shows that its contribution is small in this recording: at most, 1.4 percentage points in the reference profiles and eight non-profilable days over the recording. The readings that receive a large reconstruction variance are precisely those that are already discounted by L1, so the sample quality dominates the result. The propagation therefore acts as a safeguard that becomes relevant when repair is applied to moderately faulty readings or when more aggressive estimators, such as imputers of long gaps, are used, rather than as the main source of the observed effects. Finally, the protoforms reorient fuzzy linguistic summarization [33,37] from the monitored phenomenon, the subject of previous summaries of activity and physiological streams [38,39,41], to the trustworthiness of its description, in statements short enough to accompany a clinical report.

### 4.5. Clinical Significance

The most consequential distortions concern the low range. A TBR of 16%, as reported naïvely for Day 352, would exceed the consensus target of less than 4% [1] four-fold and could prompt a reduction in insulin, whereas the reliability-aware estimate of 3% lies within target. More generally, a report that pools days of very different reliability conveys a precision that the data do not support; identifying the days that should not be profiled, and stating which bands and periods are unreliable, would allow a clinician to discount them or to request a better-worn period before adjusting therapy. These implications must nonetheless be read with caution: they derive from a single participant, the effect of the corrected metrics on clinical decisions has not been assessed, and whether the proportion of unreliable days generalizes to other patients, sensors and wearing habits remains to be established.

### 4.6. Generalizability

The pipeline is domain-agnostic in its structure, but its transfer to another sensor rests on explicit assumptions: (i) the stream is a timestamped scalar series with a known nominal cadence Δ; (ii) the sensor has a bounded measurement range at which readings saturate; (iii) a maximum plausible rate of change ρ can be stated from the physics or physiology of the measured quantity; and (iv) the value axis can be partitioned into bands that are meaningful to the end user. Under these assumptions, the saturation, rate and missingness primitives transfer by re-parameterization, whereas stagnation and oscillation require two shape parameters each that encode knowledge of the sensor’s failure signatures, and the bands require domain-specific thresholds. Failure modes that preserve physiological plausibility, such as compression artifacts [14] or slow drift, are not captured by the five primitives and would require additional ones. Since the method has been evaluated only on CGM data, its applicability to other sensors is a hypothesis supported by its construction, not a demonstrated result.

### 4.7. Limitations

The study has further limitations. First, it rests on a single long recording; although 294 days cover a broad variety of daily conditions, the results cannot be generalized to other patients without a multi-subject study. Second, no independent reference standard was available: capillary or laboratory measurements were not collected, and the manufacturer’s cloud holds the only copy of the series. The method therefore assesses the plausibility and completeness of the data, not the accuracy of the sensor against blood glucose, which rests on the clinical validation of the factory-calibrated device and on the participant’s routine verification of its correct functioning. Third, the reference days were labeled with the same clinical knowledge that the fault rules encode, so agreement on them is a consistency check rather than an independent validation. Fourth, the fault thresholds, band boundaries and protoform templates are fixed a priori from expert rules; their shapes are reasonable but not unique. Fifth, acquisition interruptions are not logged separately and cannot be distinguished from sensor-side gaps. Finally, the phenotyping relies on *k*-means over two time-in-band features, a deliberately simple choice that captures coarse phenotypes; richer representations could reveal changes that the present clustering does not.

## 5. Conclusions

This work has shown that, in continuous monitoring, the reliability of the data is better treated as a field over the time and value range than as a single figure, and that doing so changes the knowledge derived from a sensor. In light of the results, its contributions are threefold. First, formulating reliability as a fuzzy, time- and band-conditioned quantity computed on the raw signal made it possible to establish that a day can be reliable in one glucose band and unreliable in another, such as on Days 158 and 352. Second, the five-layer pipeline, together with the acquisition architecture that feeds it, carried this judgment through to the clinical aggregates: clean days were left essentially untouched, while artifact-inflated metrics were corrected (a TBR of 16% reduced to 3% on the most corrupted day) and annotated with veracity degrees and protoforms stating which bands and periods cannot be believed. Third, over 294 days of a 14-month recording, the global veracity acted as a graded counterpart of the consensus 70% active-time rule: 38% of the days were judged to be too unreliable to profile, 5% changed phenotype, and the graded criterion retained or rejected days that the crisp rule would have treated otherwise. Rather than a single, falsely precise number, the method returns an aggregate, its veracity, and a sentence a practitioner can read. Future work will validate the pipeline in a multi-subject cohort, ideally with reference blood-glucose measurements, extend the set of primitives to plausibility-preserving faults such as compression artifacts, and test its transfer to sensing domains beyond glucose. Since the acquisition tier already enumerates every patient who authorizes its account, extending the study to a cohort is a matter of scale rather than of redesign. 

## Figures and Tables

**Table 1:** Traceable mapping of the 30 reviewed studies. Each row groups the studies that share a research objective and a pipeline layer (the right-hand flows of Figure 1).

Pipeline Layer	Objective	Domains (Studies)	References
L1 Fault tagging	Fault detection	CGM (4)	[14,15,16,17]
	Quality assessment	Health & wearable (1)	[19]
L2 Fuzzy windowing	Aggregation	Health & wearable (2), IoT (1)	[39,40,41]
	Description	Health & wearable (1)	[38]
L3 Band reliability	Quality assessment	Environmental (1), Health & wearable (1), Industrial & structural (1), IoT (2), ML & general (1)	[20,21,22,23,24,25]
L4 Routed repair	Denoising	CGM (4), Health & wearable (1)	[2,10,11,12,18]
	Imputation	CGM (1), Fuzzy soft-computing (1), Health & wearable (1), ML & general (2)	[13,26,29,30,31]
L5 Veracity aggregation	Description	Fuzzy soft-computing (5)	[33,34,35,36,37]

**Table 2:** Parameters of the CGM instantiation. Ramps (Equation (1)) are given from “doubt begins” (*a*) to “full membership” (*b*). Basis: sensor, fixed by the device specification; physiological, fixed by clinical knowledge of glucose dynamics; clinical, centered on consensus thresholds; expert rule, set a priori from expert rules on CGM failure signatures (Section 2.4); design, set by the authors.

Layer	Parameter	Value	Basis
L1	Cadence Δ	5 min	sensor
L1	Range $\left[x_{\min},x_{\max}\right]$	[39,400] mg/dL	sensor
L1	Saturation, floor/ceiling	48→39/380→400 mg/dL	sensor
L1	Rate ρ→2ρ	2→4 mg/dL·min^−1^	physiological
L1	Stagnation $\ell_{a}\to \ell_{b}$	4→10 readings	expert rule
L1	Gap 1.5Δ→6Δ	7.5→30 min	sensor (cadence)
L1	Oscillation $s_{a}\to s_{b}$	8→25 mg/dL	expert rule
L2	Window spacing $h_{c}$/width $s_{W}$	30/25 min	design
L3	Hypoglycemic band	75→65 mg/dL	clinical
L3	Hyperglycemic band	170→190 mg/dL	clinical
L4	Routing threshold $\theta_{r}$	0.3	design
L4	Running-median window	5 readings	design
L4	Reconstruction s.d. rules	Equation (18)	design
L5	$\sigma_{0}$/$\sigma_{\mathrm{ref}}$	2/10 mg/dL	design
L5	Expected readings per day *N*	288	sensor (cadence)
L5	Protoform threshold $\theta_{p}$/mass $m_{\min}$	0.35/3	design
L5	Periods of the day	Equation (21)	design
L5	Quantifiers	Equation (22)	design

**Table 3:** Per-sample quality on the ten reference days, ordered by mean quality. Character: dominant fault character according to the feedback of the participant and the physician (Section 2.5): *clean control*, no known incident; *missing*, long stretches without readings; *anomaly*, readings reported as anomalous (in the indicated band, when known); *oscillation*, physiologically impossible alternation of values; *floor*, readings clamped at the assay floor. *n*: number of readings of the day. Mean *q*: mean per-sample quality, $\frac{1}{n}\sum_{i}q_{i}$ (Equation (7)). q<0.5: number of readings with quality below 0.5, and percentage of the day’s readings. Days 90 and 184 contain fewer than 120 readings and lie outside of the eligible set (Section 2.1).

Day	Character	*n*	Mean *q*	q<0.5, *n* (%)
Day 9	clean control	275	0.99	0 (0.0)
Day 72	clean control	200	0.98	5 (2.5)
Day 52	missing	181	0.87	24 (13.3)
Day 161	anomaly (high band)	240	0.79	38 (15.8)
Day 158	anomaly (low band)	244	0.78	53 (21.7)
Day 352	oscillation + floor	239	0.68	72 (30.1)
Day 192	anomaly	254	0.66	80 (31.5)
Day 90	missing	108	0.54	49 (45.4)
Day 245	oscillation	246	0.54	110 (44.7)
Day 184	anomaly	104	0.51	51 (49.0)

## Data Availability

The glucose data analyzed in this study are openly available in the Zenodo (T1GDUJA dataset [42]). The code used to process the data and generate the results is available from the corresponding author upon request.

## Abbreviations

The following abbreviations are used in this manuscript:

API	Application Programming Interface
ARI	Adjusted Rand Index
BLE	Bluetooth Low Energy
CGM	Continuous Glucose Monitoring
HTTPS	Hypertext Transfer Protocol Secure
IoT	Internet of Things
JSON	JavaScript Object Notation
MAD	Median Absolute Deviation
ML	Machine Learning
REST	Representational State Transfer
TAR	Time Above Range
TBR	Time Below Range
TIB	Time in Band
TIR	Time in Range
TLS	Transport Layer Security
UTC	Coordinated Universal Time
